# Exploratory analysis of L1 retrotransposons expression in autism﻿

**DOI:** 10.1186/s13229-023-00554-5

**Published:** 2023-06-28

**Authors:** Giovanni Spirito, Michele Filosi, Enrico Domenici, Damiano Mangoni, Stefano Gustincich, Remo Sanges

**Affiliations:** 1 Scuola Internazionale Superiore di Studi Avanzati (SISSA), Area of Neuroscience, Via Bonomea 265, 34136 Trieste, Italy; 2 Central RNA Laboratory, Istituto Italiano di Tecnologia – IIT, Via Enrico Melen 83, Building B, 16152 Genoa, Italy; 3 CMP3vda, Via Lavoratori Vittime del Col Du Mont 28, Aosta, Italy; 4 Department of Cellular, Computational and Integrative Biology (CIBIO), University of Trento, Trento, TN Italy; 5 Eurac Research, Institute for Biomedicine, Bolzano, BZ Italy; 6 Fondazione The Microsoft Research - University of Trento Centre for Computational and Systems Biology (COSBI), Rovereto, TN Italy

**Keywords:** Autism, Transcriptomics, L1 retrotransposons expression

## Abstract

**Background:**

Autism spectrum disorder (ASD) is a set of highly heterogeneous neurodevelopmental diseases whose genetic etiology is not completely understood. Several investigations have relied on transcriptome analysis from peripheral tissues to dissect ASD into homogenous molecular phenotypes. Recently, analysis of changes in gene expression from postmortem brain tissues has identified sets of genes that are involved in pathways previously associated with ASD etiology. In addition to protein-coding transcripts, the human transcriptome is composed by a large set of non-coding RNAs and transposable elements (TEs). Advancements in sequencing technologies have proven that TEs can be transcribed in a regulated fashion, and their dysregulation might have a role in brain diseases.

**Methods:**

We exploited published datasets comprising RNA-seq data from (1) postmortem brain of ASD subjects, (2) in vitro cell cultures where ten different ASD-relevant genes were knocked out and (3) blood of discordant siblings. We measured the expression levels of evolutionarily young full-length transposable L1 elements and characterized the genomic location of deregulated L1s assessing their potential impact on the transcription of ASD-relevant genes. We analyzed every sample independently, avoiding to pool together the disease subjects to unmask the heterogeneity of the molecular phenotypes.

**Results:**

We detected a strong upregulation of intronic full-length L1s in a subset of postmortem brain samples and in in vitro differentiated neurons from iPSC knocked out for ATRX. L1 upregulation correlated with an high number of deregulated genes and retained introns. In the anterior cingulate cortex of one subject, a small number of significantly upregulated L1s overlapped with ASD-relevant genes that were significantly downregulated, suggesting the possible existence of a negative effect of L1 transcription on host transcripts.

**Limitations:**

Our analyses must be considered exploratory and will need to be validated in bigger cohorts. The main limitation is given by the small sample size and by the lack of replicates for postmortem brain samples. Measuring the transcription of locus-specific TEs is complicated by the repetitive nature of their sequence, which reduces the accuracy in mapping sequencing reads to the correct genomic locus.

**Conclusions:**

L1 upregulation in ASD appears to be limited to a subset of subjects that are also characterized by a general deregulation of the expression of canonical genes and an increase in intron retention. In some samples from the anterior cingulate cortex, L1s upregulation seems to directly impair the expression of some ASD-relevant genes by a still unknown mechanism. L1s upregulation may therefore identify a group of ASD subjects with common molecular features and helps stratifying individuals for novel strategies of therapeutic intervention.

**Supplementary Information:**

The online version contains supplementary material available at 10.1186/s13229-023-00554-5.

## Background

Autism spectrum disorders (ASDs) are a set of heterogeneous neurodevelopmental conditions mainly involving impaired communication and repetitive behaviors [1]. ASD is the most common neurodevelopmental condition in human: The median worldwide prevalence of autism is around 1% according to the latest worldwide surveys [1]. The etiology of autism presents a strong genetic component: Twin and sibling studies have consistently shown that ASD is one of the most highly heritable complex disorders in humans [2]. In the past decade, several studies exploiting whole-exome sequencing (WES) data highlighted both de novo and inherited deleterious mutations which are either causative or contributing to the autistic phenotype [3–9]. The increasing scope of WES studies and the ever-growing cohort sizes expanded the discovery rate of ASD-associated genes, to the point where up to 1000 genes are estimated to contribute to a different degree to ASD etiology and are collected in the SFARI genes database [10, 11]. Yet, none of these genes accounts for more than 1% of idiopathic ASD cases [12]. This extreme variability may be at the basis of the phenotypic heterogeneity characteristic of ASD. Hence, the identification of subgroups with a more homogeneous molecular asset is essential to comprehend ASD etiology and program personalized treatments. Understanding how the large number of genes implicated in ASD susceptibility may converge to affect human brain development is critical [12]. Similar to other neuropsychiatric disorders, most of the genes involved in ASD encode for neuronal components crucial for brain function [10, 11]. In addition, a relevant portion of genes are involved in general transcriptional regulation and/or chromatin remodeling [10, 13]. The link between deleterious mutations affecting a chromatin-regulatory gene and those involved in synaptic transmission and brain activity remains unclear, although a reasonable hypothesis suggests chromatin-regulatory gene mutations may affect transcriptional programs impacting genes involved in synaptic transmission and brain activity.

Recent works on *postmortem* brain tissues revealed shared abnormalities in gene expression in a large subset of autism cases [14–16]. Namely, two main types of gene co-expression modules were shown to be consistently deregulated: (1) downregulated genes involved in synaptic transmission, encoding for neuronal markers and enriched for ASD-associated SFARI genes and (2) modules of upregulated genes involved in immune and inflammatory responses, enriched for markers of microglia and astrocytes, but generally not for genes directly associated with ASD [14–18]. Multiple independent studies observed perturbations of epigenetic marks distribution in *postmortem* brain tissue of individuals with ASD [19–21]. Both Wong et al. [16] and Nardone et al. [19] reported an altered DNA methylation landscape among multiple brain regions of ASD individuals. Corley et al. [21] showed that epigenetic alterations detected in ASD were preferentially directed at intragenic and bivalently modified chromatin domains of genes predominately involved in neurodevelopment. Interestingly, the methylation landscape in adult neurons affected by ASD closely resembled the pattern of earlier time points in fetal brain development [21]. These findings suggest that a delay in the epigenetic program can contribute to deleterious transcriptional programs and to the establishment of ASD phenotypes. This model is supported by the identification of mutations in genes involved in the regulation of both DNA and histone methylation during brain development [13]. A recent work by Wong et al. [16] integrated data from gene expression, DNA methylation and histone acetylation from ASD and healthy individuals, proposing the existence of two major subgroups of ASD. The first subgroup recapitulated all known molecular changes typical of ASD [16]. The second one was indistinguishable from control samples in terms of transcriptional and epigenetic alterations. It is therefore tempting to stratify deleterious genomic variants by classifying their molecular effects into subgroups at higher resolution. In this scenario, a recent publication identified a set of human regulatory regions evolved after the separation from old world monkeys and highlighted how these were enriched within genomic regulatory regions altered in ASD [22]. Indeed, genomic loci relative to hominoid-specific regulatory regions showed a significant overlap with regions that lose the H3K27ac histone mark, typical of active enhancers, in ASD subjects [22]. This evidence suggests that ASD-related epigenetic defects may be caused by altered activity of evolutionarily young regulatory regions.

Transposable elements (TEs) are genomic sequences capable to mobilize [23] and shape the regulatory landscape within the host genome [24–27]. Long Interspersed Nuclear Elements 1 (L1s) are the most abundant autonomous TEs (~ 17% of the human genome) and the only transposon class known to retain the ability to mobilize autonomously in human [28]. Full-length (FL) L1 elements are about 6 kb long and take advantage of a *copy and paste* mechanism where a full-length sequence gives rise to a L1 RNA intermediate which is reverse-transcribed into a new genomic locus. FL L1s contain two open reading frames (ORF1 and ORF2), encoding for, respectively, a nucleic acid chaperone and a protein with endonuclease and reverse transcriptase activity mediating retrotransposition [28]. Retrotransposition events mostly produce 5′-truncated L1s that are unable to re-mobilize. However, L1s sequences can have an impact on the regulation of transcription of flanking genes [29, 30].

Only about 100 out of more than 10,000 full-length L1s found in the human genome [31] are potentially active [32]. To prevent potential deleterious effects of L1 abnormal activity, cells have developed several mechanisms to safeguard and fine-tune L1 retrotransposition. These include DNA methylation, transcriptional repression and L1s RNA degradation though the activity of the PIWI/piRNA pathway [33]. Nevertheless, TEs are known to escape silencing at specific embryonic stages [34], affecting early human development by regulating nearby protein-coding genes. Waves of hypomethylation during embryogenesis are linked to higher rates of transcription and retrotransposition of L1 RNAs [35]. Somatic L1 retrotransposition has been observed in the neuronal lineage leading to brain mosaicism, and L1 activity has been shown deregulated in a plethora of neurodegenerative and neurodevelopmental diseases [35–40] although its extent and functional significance remain unclear [35, 40, 41]. Recently, attention has been focused on the functional role of L1s independently from retrotransposition [42]. The majority of L1 RNAs are retained in the nucleus, and they can function as regulatory long non-coding RNAs (lncRNAs), controlling transcriptional and chromatin landscapes. For example, L1 RNAs are required for mouse embryonic stem cell (mESC) self-renewal and pre-implantation during development [43]. L1 RNA expression and mobilization must therefore be considered two independent activities under distinct regulatory pathways and with different functional outcomes [44, 45].

Altered DNA methylation levels within L1 sequences have been shown in multiple neurodevelopmental diseases, including ASD [46]. The reduction of methylation and an increase in L1 expression was reported in ASD *postmortem* brains [46, 47]. Here, trimethylation of histone H3K9 (H3K9me3), which is responsible for the formation of condensed heterochromatin and prevents L1 activation, was significantly reduced at L1 ORF1 and ORF2 sequences but not at the 5′-UTRs in ASD samples [46]. Furthermore, Tangsuwansri et al. [47] demonstrated that, in lymphoblastoid cell lines derived from a subset of ASD subjects with severe language impairment, the overall methylation level of L1 elements was decreased compared to controls, and this was inversely correlated with the level of expression of L1-containing genes [47].

In this study, we aim at quantifying the expression of young and transcriptionally active L1 elements in ASD assessing whether specific individuals affected by ASD or in vitro models of ASD show an altered L1s expression. Furthermore, we characterize and explore the transcriptional dynamics of L1 elements and the possible impact of their expression on ASD-relevant genes.

## Methods

### Datasets used

#### RNA-seq datasets

We took advantage of public data provided by Velmeshev et al. [48] (PRJNA434002). This dataset comprises *postmortem* whole-tissue bulk RNA-seq PolyA + stranded data from anterior cingulate cortex (ACC) and prefrontal cortex (PFC) and blood-derived WES data of 15 ASD cases and 16 matched controls. Since RNA-seq was performed for both tissues for only 7 out of 15 ASD individuals, while for the remaining individuals only sequencing of either ACC or PFC was performed, the two datasets are only partially overlapping. Additionally, we retrieved RNA-seq PolyA + stranded data from induced pluripotent stem cell (iPSC) and neuron cells knockout (KO) for ten ASD-relevant genes (*AFF2, ANOS1, ASTN2, ATRX*, *CACNA1C*, *CHD8, DLGAP2, KCNQ2*, *SCN2A* and *TENM1*) [49]. The dataset comprises a total of 86 RNA-seq samples, 42 from iPSCs and 44 from neurons. KOs were performed for each one of the ten genes through a CRISPR gene editing method to insert premature termination sites [49]. We analyzed RNA-seq data derived from blood of 73 pairs of discordant ASD siblings for a total of 146 samples, generated by PolyA + unstranded RNA sequencing on Illumina RNASeq Platform [50].

#### Chromatin markers

We retrieved ChIP-seq data in BigWig format from the NIH Roadmap epigenomics mapping consortium (GSM772833, GSM772834, GSM773012, GSM773013, GSM773014, GSM773015) [51]. Retrieved data were relative to ChIP-seq experiments for six major histone modification (H3K4me1, H3K4me3, H3K9me3, H3K9ac, H3K27me3 and H3K27ac) from *postmortem* mid-frontal lobe of a healthy individual. We used this data to calculate the enrichment for different histonic markers within upregulated and total FL L1HS/L1PA.

#### ATRX ChIP-seq

ATRX ChIP-seq data in BigWig format were retrieved from a recent publication (PRJNA289924) [52]. XL-MNase ChIP-seq of ATRX was performed in the erythroleukemic cell line K562, and two independent replicates using different ATRX antibodies were performed. Retrieved data are relative to the enrichment of the two replicates over the input used as a control.

### TEs and genes quantification and differential expression

**Fig. 1﻿:**
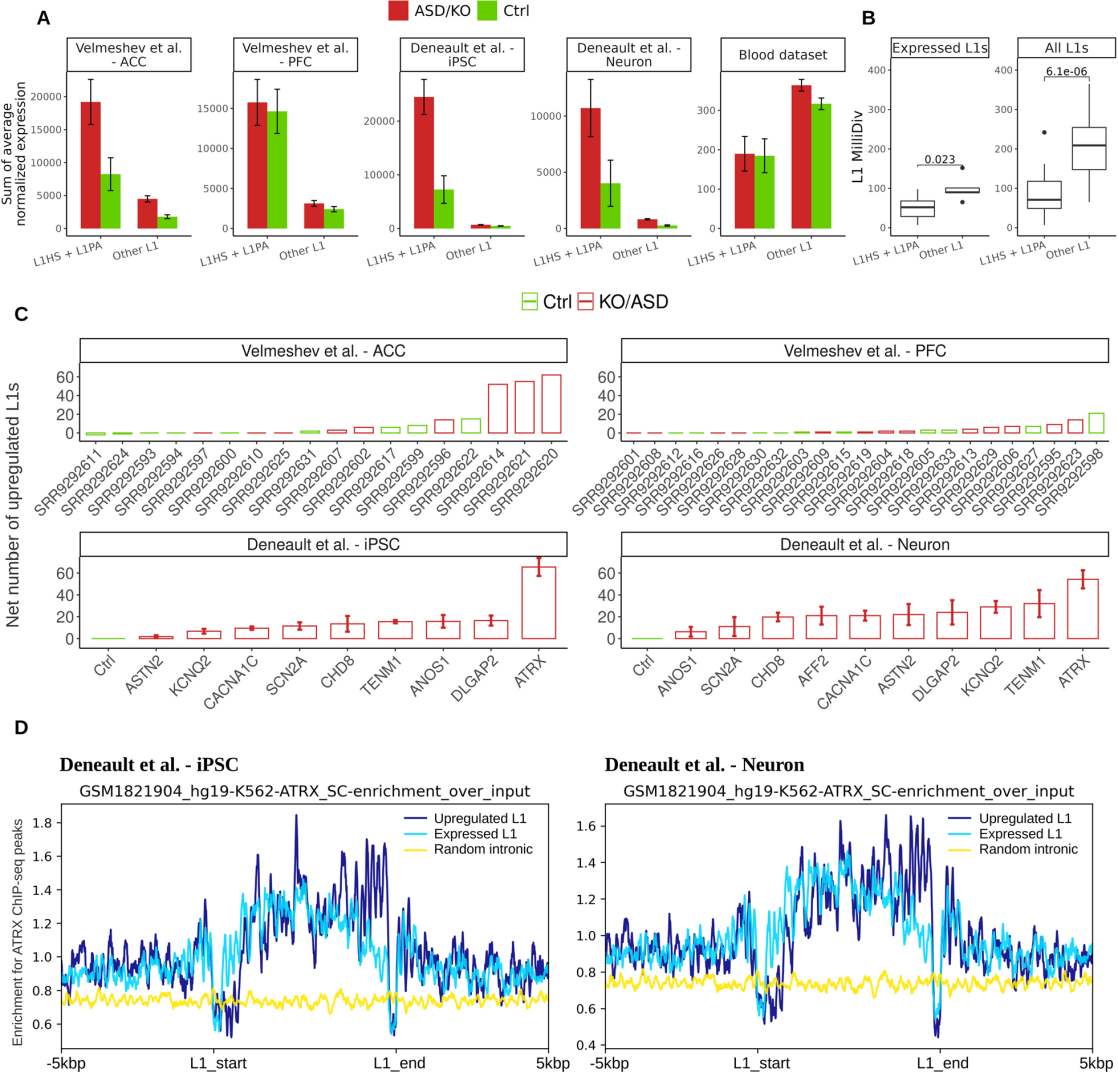
Upregulation of evolutionarily young FL L1s in ASD. **A** Sum of average normalized expression of L1 subfamilies in all samples, quantified with TEspeX. L1 subfamilies have been divided into two groups: one comprising L1HS and all 20 L1PA subfamilies and one including all other 96 L1 subfamilies, and read counts have been normalized with DEseq2. **B** Average MilliDiv values for all and expressed L1 subfamilies, retrieved from RepeatMasker. Expressed L1 subfamilies have been defined as L1 subfamilies associated with at least an average of 200 reads among all samples by TEspeX. **C** Net number of upregulated L1 elements calculated by subtracting the number of downregulated L1s (*z* < − 3) to the number of upregulated L1s (*z* > 3) for all samples, in the case of the KO gene datasets; the average and standard deviation of the net number of upregulated L1s among replicates have been plotted. **D** Enrichment profile of ATRX ChIP-seq peaks on upregulated young FL L1s, expressed FL L1s and random 6 kbp intronic genomic coordinates

Raw RNA-seq reads were aligned to the reference genome (version hg19) with STAR [53] (version 2.6, standard parameters). Gene expression was quantified with HTseq-count [54] (version 0.11.3; parameters: -f bam -r pos -t exon -i gene_name -a 10) on the BAM files obtained with STAR. The number of reads mapping to each genomic feature defined as ‘exon’ in the GTF file retrieved from gencode version v32lift37 was quantified. In order to quantify TEs expression, we used two different software: SQuIRE [55] and TEspeX [56]. The two tools employ distinct strategies to assess TEs transcriptional activity. TEspeX quantifies TEs transcription levels by counting the number of reads mapping to a reference transcriptome built merging the RepBase [31] human TEs fasta sequences and the Ensembl [57] transcript sequences containing all the human coding and non-coding annotated transcripts using STAR (v2.6.0c). Only reads flagged as primary (-F 0 × 100 parameter) and not mapping to either coding or non-coding transcripts are counted using Python scripts and Picard FilterSamReads (v2.18.4). The amount of reads mapping to each TE subfamily fasta sequence is therefore obtained. Read counts were normalized with the median of ratios method using DEseq2 [58] estimateSizeFactors function upon producing a DESeqDataSet with the matrix of expressed TE subfamilies counts and the associated metadata. The same normalization were performed for genes. In Fig. 1A, we plot the sum of the average normalized expression separately for L1s belonging to the L1HS and L1PA subfamilies and all other L1 subfamilies for ASD and control samples for each of the analyzed datasets. On the other hand, SQuIRE (software for quantifying interspersed repeat expansion) allows for the quantification of TE upon spliced alignment of RNA-seq data to a reference genome. SQuIRE is a set of computational tools which use different strategies to combine counts from uniquely mapping and multimapping reads and then generate counts for individual TE, as well as TEs by class and subfamily. Therefore, using SQuIRE it has been possible to analyze transposable element expression on both subfamily and locus-specific (single copies of TEs annotated on RepeatMasker) level. SQuIRE (v0.9.9.92) was used. We performed an analysis which allowed us to estimate the amount of DE genes and of full-length L1HS/L1PA in each individual sample. Firstly, for each dataset, only L1HS/L1PA longer than 5 kbp and featuring at least an average of 200 reads mapped and only protein-coding genes with at least 15 reads mapped were selected. Raw read counts were normalized with DEseq2 [58]. Then, for each ASD/KO sample, we calculated a Z-score for each gene and L1 by calculating the difference between each normalized expression value and the average normalized expression of control samples and dividing the result for the standard deviation of control samples. In the case of controls, for each dataset, we iteratively compared each control sample with the distribution of all other controls. Full-length L1HS/L1PA and genes with a |Z-score|> 3 were considered differentially expressed. In the case of the Deneault et al. dataset, we also performed a standard differential gene expression analysis with DEseq2 (release 3.12). Read counts were normalized with the median of ratios method using DEseq2 estimateSizeFactors function upon producing a DESeqDataSet with the counts matrix. DEseq2 analysis was performed with standard parameters on the raw gene counts assessed by HTseq. Only protein-coding genes featuring an average of at least 200 mapped reads among the all the samples were considerate suitable for differential expression analysis. Genes with an FDR < 0.05 and LogFoldChange > 0.5 or < 0.5 were considered as differentially expressed.

### Reads mappings metrics

For each sample of the Velmeshev et al. dataset, we report the total number of mapped reads, the total number of reads labeled as mapping to no_feature, ambiguous, too_low_aQual and mapping to exons according to HTseq [54]; the total number of reads mapping to extragenic and intronic regions. HTseq defines as ‘no_feature’ reads (or read pairs) which could not be assigned to any transcript annotate in the genome; ambiguous are reads (or read pairs) which could have been assigned to more than one feature and hence were not kept into account; too_low_aQual are reads (or read pairs) which were skipped due to an alignment quality lower than 30. The number of reads mapping within introns was calculated by combining bedtools intersect and bedtools coverage [59]. We determined intronic regions by subtracting the genomic coordinates of exons to the genomic coordinates of genes with bedtools subtract [59]. We used the flag -F 1 in order to only count reads mapping inside an intron interval for 100% of the read length, and the -split flag which splits reads which are mapped across splice junctions. We used the same process to calculate the number of extragenic reads. Extragenic coordinates were obtained by subtracting the genomic coordinates of all genes with the genomic coordinates of chromosomes with bedtools subtract [59].

### ChIP-seq data enrichment profile

We computed enrichment profiles from ChIP-seq derived data using DeepTools [60]. Data were retrieved in BigWig format. Enrichment profiles were computed for genomic segments of interest using DeepTools computeMatrix and plotProfile utilities.

### Intron retention analysis

In order to estimate the rate of intron retention (IR) in groups of samples showing upregulation of L1s, we used IRFinder [61]. We built the reference for human genome GRCh37 and run IR quantification in fastq-mode, as outlined in the user manual. This mode outputs an end-to-end IR measurement upon aligning fastq reads to the built reference with STAR. Differential intron retention analysis between the group of 3 samples showing L1 upregulation and the controls has been performed with the generalized linear model (GLM), as suggested for groups of samples with more than 3 replicates per condition. Introns have been considered differentially retained if featuring an FDR < 0.05. Also, the number of retained introns in each sample was counted according to the following filters: IRratio ≥ 0.1 and IntronDepth ≥ 3 and Warnings = ‘–’ and no overlap with known features.

### L1 exonization analysis

We assessed whether upregulated intronic L1s correspond to exonized elements. Exonized TEs are TEs copies or fragments whose sequence is transcribed as a functional part of other mature canonical coding/non-coding genes. We used a custom method to estimate the percentage of fragments of the same read pair mapping to two distinct genomic *loci*. Briefly, for each dataset, all reads mapping within the boundaries of a genomic *locus* were extracted from the bam file of all samples (only properly paired mapped reads, minimum mapping quality: 30). Then, the ID of these reads was used to extract all fragments belonging to these read pairs from the same bam files; these are the ‘total fragments.’ Consequently, the coordinates of the total fragments were intersected with the genomic coordinates of a second genomic *locus*. Finally, the percentage of the read IDs found on both *loci* on the number of total fragments was calculated. The higher the percentage, the higher is the number of reads (or read pairs) spanning multiple regions of the same mRNA. We performed this analysis for three kinds of *loci* pair: (a) 1000 random expressed exons with their closest exon; (b) upregulated FL L1HS/L1PA with their closest exons; (c) 1000 random 6 kbp sequences randomly selected within the boundaries of expressed genes with their closest exons.

### L1Base2 overlap analysis

In order to match our set of expressed L1s with the L1s retrieved from L1Base2, we overlapped the genomic coordinates of our expressed L1s with the coordinates of L1Base2. We retrieved the genomic coordinates of three subgroups of L1s from the L1Base2 database [62]. In this database, full-length L1 elements in the human genome are classified as Full-Length, Intact LINE-1 Elements (FLI-L1, 146 elements), ORF2 Intact LINE-1 Elements (ORF2-L1, 107 elements), Full-Length non-Intact > 4500 bp LINE-1 Elements (FlnI-L1, 13,418 elements). The first two groups are considered to be still potentially coding for the whole L1 element (FLI-L1) or only for the ORF2 (ORF2-L1), while the third and more numerous group (FlnI-L1) is considered non-coding because of mutations not allowing the maintenance of any open reading frame. We exploited the liftover utility of the UCSC genome browser [63] to convert the genomic coordinates of the L1s retrieved from L1Base2 from GRCh38 to the GRCh37. 125 out of 13,418 FlnI-L1, 1 out of 107 ORF2-L1 and 1 out of 146 FlnI-L1 failed the conversion process and were therefore discarded from the analysis.

### Enrichment analysis of anticorrelated genes

We counted the number of overlaps between genes overlapping upregulated L1s and characterized by a negative Z-score compared to controls and (a) SFARI genes; (b) genes showing a high expression level in the CNS; and (c) genes specifically expressed in distinct cell types characteristic of the human central nervous system. SFARI genes were retrieved from the SFARI gene database (01/11/2022 release) [11]. A list of genes showing high expression level in the adult human brain was retrieved from Leblond et al. [64]. Cell-type-specific gene expression has been estimated by McKenzie et al. [65]. For this analysis, we used five sets of cell-specific genes comprising the 1000 genes with the highest fold change across two datasets of cell-specific gene expression [66, 67], relative to five cell types (neuron, oligodendrocyte, endocyte, astrocyte, microglia). We counted the number of overlaps between anticorrelated genes and each set of genes and compared it to the number of overlaps between anticorrelated genes and 1000 equally sized random sets of expressed genes used to calculate the *z* scores. We considered as significant the enrichments associated with a *z* score above 3.

### Enrichment analysis of genomic FL L1s

To assess whether annotated genomic FL L1s are more likely to be found embedded in ASD-related genes and genes specifically expressed in distinct cell types compared to all other human genes, we retrieved the genomic coordinates of all FL L1HS/PA (length > 5000) annotated in the human genome (hg19 assembly) resulting in 9077 elements. We then overlapped the genomic coordinates of the selected L1s with the genomic coordinates of SFARI genes and the previously described lists of cell-type-specific genes produced by McKenzie et al. [65]. We compared the number of overlaps found with the number of overlaps between L1s and 1000 equally sized sets of random genes.

## Results

### Expression of evolutionarily young L1 subfamilies is detected in *postmortem* brains, in in vitro models and in blood.

L1 transcriptional deregulation is observed in multiple human disorders including ASD [46, 47], through mechanisms that are not yet well understood. To explore the potential impact of L1 elements in ASD, we assessed whether and which L1 subfamilies are expressed in samples and experimental models of ASD. We retrieved public bulk RNA-seq data from *postmortem* ASD brains (Velmeshev et al. dataset [48]). The dataset consists of a total of 31 individuals (from 15 ASD donors and 16 controls) with expression profiling of anterior cingulate cortex (ACC) and prefrontal cortex (PFC). Data from both tissues were available for 10 individuals (7 ASD and 3 controls), while for the remaining 21 individuals (8 ASD and 13 controls) expression data were produced from either ACC or PFC. Therefore, analyses were performed on a total of 41 RNA-seq derived samples, 18 for ACC (9 ASD and 9 controls) and 23 for PFC (13 ASD and 10 controls) (Additional file 1: Tables S1, S2). We also retrieved RNA-seq data from induced pluripotent stem cell (iPSC) and neuronal cells knockout (KO) for ten ASD-relevant genes (*AFF2, ANOS1, ASTN2, ATRX*, *CACNA1C*, *CHD8, DLGAP2, KCNQ2*, *SCN2A* and *TENM1*) (Deneault et al. dataset, [49]). The dataset comprised a total of 87 RNA-seq samples, 42 from iPSCs and 45 from neurons. KOs were performed for each ASD-relevant gene through a CRISPR gene editing method to insert premature termination sites (Additional file 1: Tables S1, S3). Furthermore, we used RNA-seq data from whole blood of 73 pairs of discordant ASD siblings for a total of 146 samples (Additional file 1: Tables S1, S4).

FASTQ files from each sample were mapped on the human genome (Additional file 1: Table S5). To quantify L1 expression, two softwares were used: TEspeX [56] and SQuIRE [55]. TEspeX measures TEs expression not counting reads possibly generated from TE fragments embedded in the exons of annotated canonical transcripts. It cumulatively quantifies the expression of TE subfamilies annotated in RepeatMasker [31] by counting the sequencing reads mapped to the consensus sequence of each TE subfamily. SQuIRE [55] allows to count reads mapped to each genomic *locus* where a TE is annotated. Both tools should enrich for the uniquely mapped reads; however, a portion of multimapping reads is likely taken into account for quantification. We measured the expression of a total of 117 human L1 subfamilies. First, we used TEspeX to determine which L1 subfamilies are more expressed independently from the specific host transcripts and genomic *locus*. We then exploited SQuIRE to pinpoint which specific L1 fragments belonging to the most expressed subfamilies are transcribed at the *locus*-specific level. For each dataset, we defined as expressed all L1s fragments with an average of at least 200 reads mapped among all samples. According to our analysis, L1HS was the L1 subfamily with the highest expression level in all datasets, followed by several L1PA subfamilies (Additional file 2: Table S6). Figure 1A shows the sum of the average normalized expression of specific grouping of L1 subfamilies. In ASD brain samples, KO cell lines and all their controls, L1HS and L1PA subfamilies made up ~ 80–90% of the total normalized L1 expression, while the other L1 subfamilies cumulatively showed a modest level of expression (Fig. 1A, Additional file 2: Tables S6, S7). On the other hand, L1HS and L1PA subfamilies constituted only about 35–40% of the total normalized L1 expression in the blood dataset, for both ASD and controls (Fig. 1A).

Evolutionarily young TEs are expected to show a lower number of mutations which might increase their probability to be transcribed compared to older TEs. A measure of the evolutionary age of TEs is measured as base mismatches in parts per thousand (MilliDiv) with respect to its consensus sequence. We retrieved MilliDiv values for each L1 fragment annotated in the human genome from RepeatMasker [31] and calculated the average MilliDiv for each subfamily. As expected, the average MilliDiv for L1HS and L1PA fragments was significantly lower compared to that of the fragments of other L1 subfamilies and, interestingly, the average MilliDiv for the group of expressed L1 elements was lower than the corresponding one of the same subfamily containing also the non-expressed ones (Fig. 1B). We therefore decided to focus our analyses on L1HS and 20 L1PA subfamilies.

### L1 elements are upregulated in *postmortem* brains from a subset of ASD individuals and ASD model samples

To assess whether any of the analyzed ASD sample would show altered expression of young L1 elements, we measured the expression of L1s annotated in the human genome in all samples with SQuIRE [55]. We then performed L1-related differential expression analysis comparing ASD samples *versus* controls. To reduce the risk of quantifying the expression of small exonized L1 fragments whose expression can be a consequence of the transcription of their host gene, we limited our analysis on likely full-length (> 5 kbp) fragments showing an average of at least 200 reads mapped among samples of each dataset and belonging to L1HS and L1PA subfamilies. A total of 128,506 L1HS and L1PA fragments are annotated in RepeatMasker [31]; of these 9077 are more than 5 kbp long. From SQuIRE analysis, we classified 100 L1s from Velmeshev et al. and 175 L1s from Deneault et al. as expressed (> 200 average reads mapped among all samples) (Additional file 3: Tables S8, S9). Of them 36 were commonly expressed in both datasets.

We then performed L1 expression analyses on each single individual. Briefly, the number of reads mapped to each L1 fragment was first normalized with DEseq2 [58]. To infer for differential expression in each single individual, in the absence of replicates, we calculated a *z* score for each L1 fragment comparing its expression level in each sample against the distribution of its expression levels in the group of controls. We therefore chose to analyze every single subject independently, avoiding to aggregate the disease subjects and considering them as a group of replicates. The reason behind this choice relies on the fact that ASD cases represent a heterogeneous cohort and aggregate analyses often tends to mask the heterogeneity of the molecular phenotypes. Although this analysis allows the identification of specific patterns in single subjects, results should be considered as exploratory and to be validated on bigger cohorts because *z* score analysis is more powerful with bigger sample sizes. For each sample, we classified as potentially upregulated those L1 fragments characterized by a *z* score higher than 3, and as potentially downregulated those L1 fragments characterized by a* z* score lower than -3. We detected a total of 2196 cases of expressed L1 upregulation and 315 cases of downregulation in the analyzed samples (Additional file 3: Table S10). We then calculated the net number of expressed fragments by subtracting the number of downregulated L1s to the number of upregulated L1s for each sample. Overall, ASD brain and KO samples always showed a positive net number of expressed L1s compared to controls. In most control samples, the net number of expressed L1s was either null or very low but positive, except for two control samples of the Velmeshev et al. ACC dataset, which showed a moderately negative value (Fig. 1C). In the case of the Velmeshev et al. dataset, three ASD samples in the ACC showed a considerably higher amount of L1 net expression compared to all other samples. These samples showed a net number of about 60 upregulated L1s, while all other samples did not present more than 25 net potentially upregulated L1s (Fig. 1C). On the other hand, no PFC sample showed a substantially increased number of upregulated L1s compared to the other samples. Furthermore, the three samples characterized by strong L1 upregulation (SRR9292614, SRR9292620, SRR9292621) when compared to all other samples in ACC did not show the same trend in their PFC counterpart (SRR9292613, SRR9292619, SRR9292623). Therefore, the observed L1 upregulation probably occurs only in specific areas of the brain.

In order to rule out the possibility that these results could arise from quality or technical biases specific of the three significant samples (SRR292614, SRR292620, and SRR292621), we calculated several metrics based on reads mappings (Additional file 1: Table S5) and evaluated the* z* score for each set of normalized reads in order to detect potential mapping outliers. Samples SRR292614, SRR292620 and SRR292621 did not show any significant* z* score in any of the metrics suggesting that the results associated with these samples were not resulting from potential biases. These samples showed a relatively high and positive* z* score related to the number of reads mapping within introns, with the sample SRR9292620 showing a statistically significant positive* z* score. Rather than representing a technical artefact, in the absence of other outlier measures, we believe this result could indicate a potential alteration of splicing that will be further discussed in the remaining part of this study.

In the Deneault et al. dataset, both iPSC and differentiated neurons KO for ATRX showed a substantially higher average net number of expressed L1s (about 60) compared to the other KOs, which never exhibited a value higher than 30 (Fig. 1C). Of note, in all KO samples the average net number of expressed L1 compared to controls was positive. We interpret this result as an indication of L1 upregulation, suggesting that the loss of function of ASD-related genes might lead to an increase in L1 transcriptional activity.

To determine the magnitude of differential expression related to L1s, we calculated the log2 ratio between the normalized expression of each L1 in each sample and the average normalized expression of controls, separately for each sample of each dataset analyzed (Additional file 3: Table S10). The average log2 fold change of DE L1s is about 1.2 (2.3-fold on linear scale), median 1.0 (twofold on linear scale). We also retrieved the genomic coordinates of three subgroups of L1s from the L1Base2 database [62] and calculated the overlap of the genomic coordinates of expressed L1s with the coordinates of the L1Base2 annotations (see ‘Methods’ section). 97 out of 100 L1s expressed in the Velmeshev et al. dataset and 162 out of 175 expressed L1s in the Deneault et al. dataset matched with the FlnI-L1 (full-length non-intact) set. Only 1 L1 expressed in the Deneault dataset matched with the ORF2-L1 group and 1 from the Velmeshev dataset and 6 from the Deneault dataset matched with the FLI-L1 (full-length intact). Overall, more than 90% of the expressed L1s overlap elements in the L1Base2 database and can be considered full length, albeit only about 5% of the expressed L1s retain its coding potential. These results suggest that the potential cellular effects of L1 dysregulation may occur mostly at the RNA level.

Finally, we performed the same L1 expression analyses on a total of 146 RNA-seq whole blood samples derived from individuals affected by ASD and their unaffected siblings. However, no L1 fragment showed a* z* score above 3 in any ASD or control sample. We then plotted the cumulative expression of L1 subfamilies quantified with TEspeX. Overall, all blood samples (ASD and controls) showed similar total L1HS/L1PA expression levels (Additional file 4: Fig. S1A). Furthermore, the distribution of the ratio of the total L1HS and L1PA normalized expression between each pair of discordant siblings (ASD/Control) showed no alterations of L1 levels in the blood of discordant siblings (Additional file 4: Fig. S1B).

Taken together, these results suggest that only a subset of ASD subjects may present a strong increase in the transcriptional activity of young full-length L1 elements, probably in specific brain regions.

### Evaluation of L1 expression in the context of mutated genes

iPSCs and differentiated neurons KO for ATRX displayed a pervasive upregulation of young FL L1 elements, while the KO of nine other ASD-relevant genes led to a more limited increase of L1 expression. Hence, we speculate that the loss of function of specific genes may differently affect L1 transcriptional regulation. ATRX is a SFARI gene that encodes for a protein which contains an ATPase/helicase domain belonging to the SWI/SNF family of chromatin remodeling proteins [52, 68]. It has been demonstrated that ATRX knockout in mouse ES cells causes increased chromatin accessibility at genomic loci occupied by retrotransposons [68] suggesting that this gene has a direct role in the establishment and maintenance of heterochromatin also at the level of L1s. To validate this hypothesis, we retrieved ChIP-seq data from a recent publication [52], where the authors performed a comprehensive ChIP-sequencing on ATRX in human cell lines. To assess whether ATRX may specifically act on genomic regions occupied by the FL L1s that were upregulated in our analysis, we computed a profile of ATRX ChIP-seq data separately on the genomic coordinates of (1) the expressed and (2) the upregulated L1 *loci* compared to controls L1HS/L1PA, as well as of (3) one thousand of 6 kbp long genomic coordinates randomly selected within annotated introns as controls. The profile was computed with DeepTools [60] computeMatrix utility and plotted with plotProfiles. In both iPSC and neurons, expressed and upregulated L1HS/L1PA showed a binding enrichment compared to both their up/downstream boundaries and to random intronic segments (Fig. 1D). Interestingly, the upregulated L1s showed a decrease of ATRX binding in the 5’UTR and a corresponding increase in the binding at the 3’UTR when compared to the set of expressed L1s. This binding pattern has been previously observed in correspondence of zinc finger genes and it warrants further investigation [52]. Together with the strong L1 upregulation observed in ATRX KO cells, these results add support to the fact that ATRX loss of function might lead to an increased transcription of young FL L1 elements.

### Transcriptome-wide deregulation in ASD *postmortem* brain and in vitro differentiated neurons correlates with L1 upregulation

**Fig. 2:**
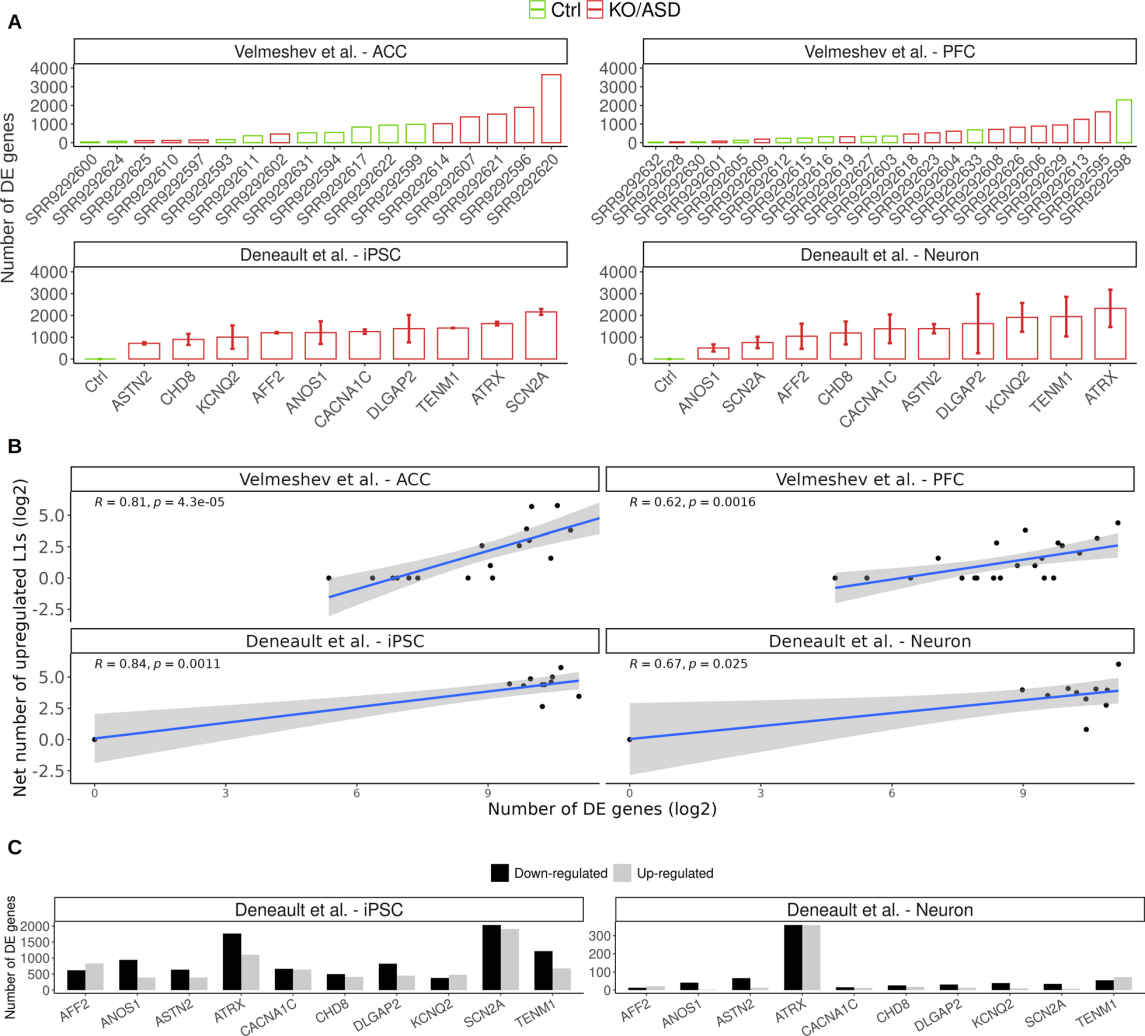
Transcriptome-wide level of deregulation in ASD brain and in vitro neurons correlates with L1 upregulation. **A** Total number of dysregulated genes (|*z* score|> 3) for all samples. In the case of the gene KO datasets, the average and standard deviation of the number of dysregulated genes among replicates has been plotted. **B** Correlation between the number of DE genes and the number of upregulated L1 in log2. **C** Number of DE genes (FDR < 0.05, |LogFoldChange|> 0.5) resulting from DEseq2 differential expression analysis

Altered gene expression is currently considered as one of the main molecular manifestations of ASD [14–16, 18]. Since in our analysis a specific subset of ASD samples showed strong L1 upregulation in independent datasets, we investigated whether altered L1 expression was associated with gene expression alteration. To define differentially expressed (DE) genes, we used the same approach applied to FL L1HS/L1PA fragments, which allowed us to characterize each subject individually in comparison with controls in absence of replicates. We applied this method only to genes associated with an average number of mapped reads above 200 across samples of each dataset. We refer to this subset of genes as expressed genes. Genes were considered DE if associated with a *z* score higher than 3 or lower than -3. Overall, the number of DE genes was not homogeneous among samples, with a subset of ASD samples showing a number of DE genes very close to controls (Fig. 2A). However, samples showing stronger L1 upregulation were among the samples with the higher number of DE genes, especially in *postmortem* brain and in vitro differentiated neurons demonstrating a significant correlation between L1 expression and the number of differentially expressed genes in the ACC dataset (Fig. 2A, B). For example, sample SRR9292620 was associated with both the highest number of net expressed L1s and with the highest number of DE genes. Furthermore, the top six samples of the Velmeshev et al.—ACC dataset with the highest L1 net expression, were at the same time the six samples with the highest number of DE genes. Similarly, in vitro differentiated neurons that were KO for ATRX, TENM1, KCNQ2 and DLGAP2 showed the highest L1 net expression and gene dysregulation.

In order to further explore this result, we carried out gene DE analysis with DEseq2 [58] by exploiting the technical replicates of KO iPSC and neuronal cell lines. To this end, the number of DE genes was plotted for all KOs (FDR < 0.05, |Log2FoldChange|> 0.5). While we observed transcriptional deregulation for all KOs in iPSCs, only KO samples for ATRX showed a considerable number of DE genes in differentiated neurons (Fig. 2C). Given that the very same samples showed the strongest L1 RNA upregulation, consistently with what we observed in the *z* score analysis, these results suggest that FL L1HS and L1PA elements might be associated with gene deregulation in mature neurons of ASD subjects.

### Upregulated L1 elements are mainly expressed within introns of actively transcribed genes

**Fig. 3:**
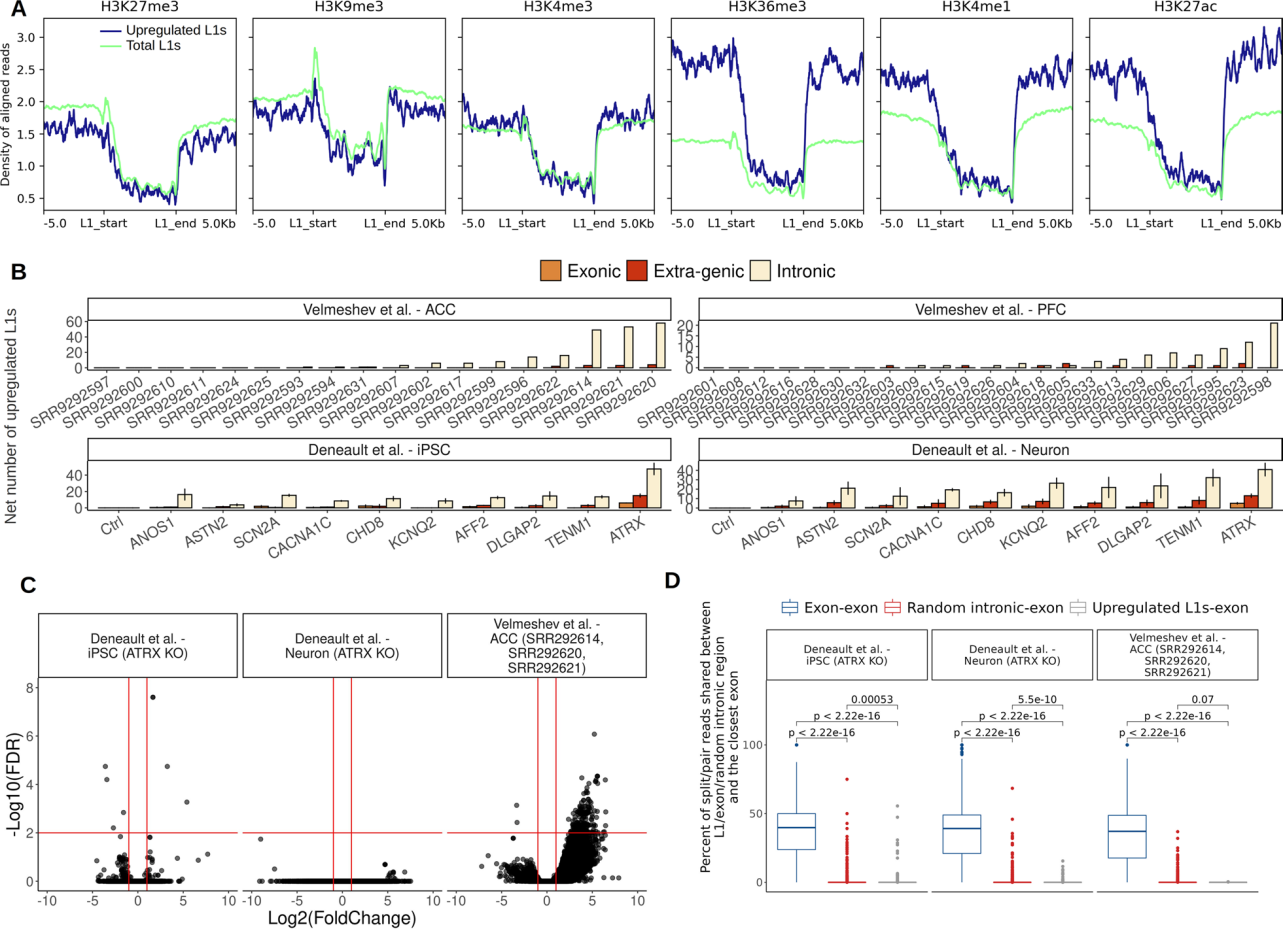
Upregulated L1 elements are expressed within introns of actively transcribed genes.** A** Histone marks aligned reads density profiles computed for all upregulated L1 elements and total annotated L1 elements for six major histone marks, the profile extends along the whole length of the L1 elements (~ 6 kbp) as well as along 5 kbp upstream and downstream with respect to each L1 element. **B** Genomic localization of all upregulated L1 elements in all samples. **C** Volcano plot representing differentially retained introns between controls and samples characterized by a strong L1 upregulation, significance lines: FDR = 0.05, |Log2(FoldChange)|= 1.** D** Percentage of reads shared among random exons and their closest upregulated L1, exons and their closest exon, random intronic 6 kbp regions and their closest exons

Given the original observation that gene deregulation was generally stronger in *postmortem* brain tissue and in vitro differentiated neurons showing L1 upregulation, we explored the genomic context of upregulated L1s and assessed the relationship between their transcription and the host gene transcripts. Firstly, we analyzed ChIP-seq data from the NIH Roadmap Epigenomics Mapping Consortium [51], focusing our attention on six major histone modifications (H3K4me1, H3K4me3, H3K9me3, H3K36me3, H3K27me3 and H3K27ac) from *postmortem* mid-frontal lobe of a control individual. Upon retrieving ChIP-seq data in wig format, and converting them to bigwig using USCS wigToBigWig utility, we computed a profile of histone mark mapping density relatively to (1) the genomic coordinates of a unique list of FL L1s upregulated in all samples and (2) of all FL L1HS/L1PA annotated in the human genome. The profile was computed with DeepTools [60] ⁠ computeMatrix utility and plotted with plotProfiles. Overall, all histone marks profiles showed a substantial drop at the level of L1 genomic coordinates, which may be due to the typically lower mappability of repetitive regions. However, upregulated L1s showed a slightly weaker enrichment for transcriptional repressive marks H3K9me3 and H3K27me3 compared to all annotated FL L1HS/L1PA, both within the boundaries of the L1 fragment and their flanking upstream and downstream regions. Interestingly, upregulated L1s exhibited a considerably stronger enrichment for activator marks (H3K36me3, H3K4me1 and H3K27ac) at the level of their flanking regions (Fig. 3A). While further analysis is needed on samples from different brain regions, these results are consistent with the model of a genomic environment more permissive to transcription for the upregulated L1s with respect to the genomic average.

We also measured the percentage of upregulated, expressed and total annotated FL L1PA/L1HS which overlap expressed genes. In both datasets, the percentage of upregulated and expressed L1s found within expressed genes greatly exceeded the percentage of total L1s. However, upregulated L1s were neither enriched nor depleted within expressed genes compared to non-deregulated or expressed L1s (Additional file 4: Fig. S2). We then overlapped the genomic coordinates of upregulated L1s with the genomic coordinates of exons and introns of canonical coding and non-coding genes retrieved from UCSC. All L1s not overlapping with either introns or exons were considered intergenic. For the vast majority of samples in all datasets, the number of upregulated intronic L1s greatly exceeded the number of upregulated exonic and intergenic L1s, especially in the samples with the highest number of upregulated L1s (Fig. 3B). These results suggest that upregulated L1s were enriched within introns of actively transcribed genes. We did not find any enrichment of a specific strand orientation preference between the L1s and the host genes. Further analysis on larger cohorts is needed to properly address the question whether a specific strand orientation combination is important for L1/host gene transcription.

Given that the majority of upregulated L1s are intronic, we wondered whether their upregulation might be a technical artefact caused by an increased intron retention. We therefore performed a differential intron retention analysis focused on the groups of samples characterized by L1 upregulation compared to controls using IRFinder [61]. While we detected only a few differentially retained introns in iPSC and neurons KO for ATRX, we detected 5 downregulated and 806 upregulated introns in the group comprising samples SRR292614, SRR292620 and SRR292621 (Fig. 3C). However, only one of these significantly upregulated retained introns overlapped an upregulated L1, suggesting that intron retention is not at the basis of the observed L1s upregulation. This lack of overlap was also confirmed by a per-sample analysis. In this case, we first identified retained introns in each sample using a specific filter on the IRFinder output and then we overlapped the identified retained introns with the upregulated L1s identified with the *z* score method. Again, also in this analysis no evidence of overlaps was found between any retained intron and upregulated L1 (Additional file 5: Table S11). Intron retention was therefore strongly increased in the brain of ASD subjects showing upregulation of L1s and general deregulation of canonical genes. This interesting pattern of expression deserves further studies to understand its functional significance and regulatory mechanism.

We then tested whether intronic upregulated L1s would be spliced into novel transcripts together with nearby annotated exons. To this end, we counted, for all groups of samples characterized by L1 upregulation, the percentage of mapped reads whose fragments are shared by pairs of genomic segments comprising: (1) a random expressed exons and its closest expressed exon, (2) an upregulated L1 and its closest exon, (3) a random 6 kbp long sequence sampled from introns of expressed genes and its closest exon. The distribution of the percentage of shared reads between upregulated L1s and their closest exon was similar to the one of random intronic segments, and significantly lower than the average percentage of shared reads between pairs of neighboring exons (Fig. 3D).

Taken together, these results suggest that upregulated L1s are mostly located within introns of expressed genes and might be expressed as independent transcriptional units.

### Upregulated L1 elements may be associated with downregulation of a small set of ASD-related host genes

**Fig. 4:**
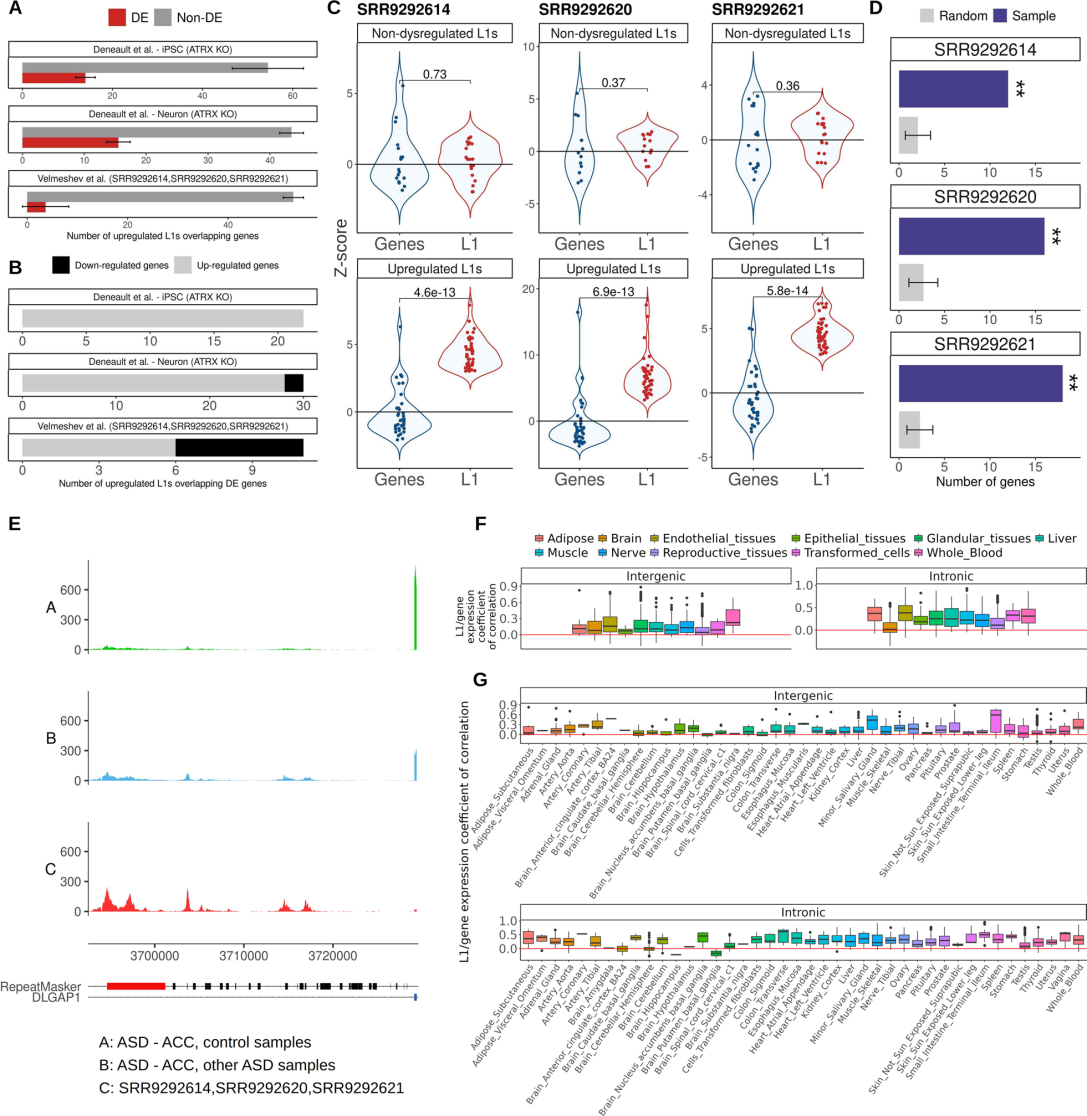
Upregulated L1 elements may negatively impact the expression of specific ASD-related host genes. **A** Number of upregulated L1 elements overlapping DE and non-DE genes. **B** Number of upregulated L1 elements overlapping upregulated and downregulated genes. **C** *z* score associated with upregulated L1s (*z* score > 3) and their overlapping genes in samples, and non-upregulated L1s (|*z* score < 2|) and their overlapping genes in samples SRR9292614, SRR9292620 and SRR9292621. **D** Number of genes overlapping upregulated L1s and associated with a negative *z* score in common with genes nearby genomic loci where the L1 RNA binds genomic DNA in mouse, compared to random distributions computed with expressed genes, **z score > 5. **E** Example of an upregulated L1 intronic to a gene showing an average negative *z* score. The sashimi plots represent the normalized expression at the level of the intronic upregulated L1 and the closest exon belonging to the DLGAP1 gene. The upregulated L1 is in red in the RepeatMasker track. Other annotated repeats are in black. DLGAP1 exon is in blue. **F** Distribution of correlation coefficients between intronic and extragenic L1s and the closest/overlapping gene for all L1/gene couples in different human tissues grouped together and **G** taken ungrouped

Starting from the model that intronic upregulated L1s might be expressed as independent transcriptional units, we assessed how they relate with the expression of their host genes. We limited our analysis to the samples with the highest L1 upregulation: SRR292614, SRR292620 and SRR292621 from *postmortem* ACC (Velmeshev et al. dataset), and iPSC and differentiated neurons KO for ATRX (Deneault et al. dataset). To this end, we counted the number of upregulated L1s found within the genomic *loci* of DE genes for each sample. We considered as DE those genes and L1s featuring a* z* score above 3 or below -3. Overall, most upregulated L1s were not found within DE genes (Fig. 4A) with the exception of 11 significantly upregulated L1s in *postmortem* ACC that were located within significantly differentially expressed genes. Of note, 5 of them (MARK1, MAPK8, OPCML, DLGAP1 and ZNF780B) resulted from a single subject and showed an upregulated L1 overlapping a downregulated gene (Fig. 4B, Table 1). Taking into account both the Velmeshev and the Deneault dataset, we identified a total of about 50 overlaps between L1 and genes in which both of them were significantly upregulated. Albeit an interesting topic to be further inspected, the concomitant increased expression of an L1 and its host gene could result from general chromatin relaxation. On the other hand, even if deriving from a single sample, we were intrigued by the 5 upregulated L1s located inside downregulated genes. This relationship led us to speculate that, in some *loci*, L1 upregulation might have a negative impact on the expression of their host genes. To further explore this possibility, we selected all intronic upregulated L1s (cutoff on *z* score > 3 for the L1s) in each of the three ACC sample displaying strong L1 upregulation separately. For each of the three samples, we then associated the* z* scores of each intronic upregulated L1 with the *z* score of their overlapping genes (without a specific cutoff on the *z* for the genes). In all the three samples, the* z* score associated with genes overlapping upregulated L1s was mostly negative, while genes overlapping non-upregulated L1s did not show any trend (Additional file 5: Tables S12–S14, Fig. 4C). This result added support to the possibility that L1 upregulation might have a negative impact on the transcription of host genes in a subset of *loci*.

**Table 1:** Differentially expressed genes overlapping upregulated L1s in specific samples

L1 chr	L1 start	L1 end	L1 ID	Gene name	Gene alteration	Sample ID
chr11	14799221	14805319	L1PA5:L1:LINE	PDE3B	Upregulated	SRR9292614
chr1	68553278	68559388	L1PA6:L1:LINE	WLS	Upregulated	SRR9292620
chr11	14799221	14805319	L1PA5:L1:LINE	PDE3B	Upregulated	SRR9292620
chr11	132190054	132196077	L1PA4:L1:LINE	NTM	Upregulated	SRR9292620
chr5	139895099	139900527	L1PA3:L1:LINE	ANKHD1-EIF4EBP3	Upregulated	SRR9292620
chr1	220814954	220821404	L1PA10:L1:LINE	MARK1	Downregulated	SRR9292620
chr10	49580263	49585413	L1PA7:L1:LINE	MAPK8	Downregulated	SRR9292620
chr11	132626675	132633167	L1PA7:L1:LINE	OPCML	Downregulated	SRR9292620
chr18	3695243	3700586	L1PA7:L1:LINE	DLGAP1	Downregulated	SRR9292620
chr19	40543822	40549961	L1PA4:L1:LINE	ZNF780B	Downregulated	SRR9292620
chr5	139895099	139900527	L1PA3:L1:LINE	ANKHD1-EIF4EBP3	Upregulated	SRR9292621

A recent publication [69] showed that, in mouse ESCs, L1-enriched genes were transcriptionally silenced by a not yet well understood mechanism which involves the transcription of the L1 element and the subsequent binding of the L1 RNA to the DNA at the level of specific *loci*. Indeed, through chromatin isolation by RNA purification (ChIRP) experiments, the authors identified ~ 25,000 genomic *loci* where the L1 RNA binds genomic DNA [69]. A subset of them were found in close proximity to a total of 2400 genes (distance < 5 kbp). Interestingly, in all three ACC samples genes overlapping upregulated L1s and associated with a negative *z* score were enriched within the human orthologs of genes nearby L1-ChIRP peaks when compared to a random distribution computed with equally sized sets of expressed genes (Fig. 4D, z score > 5). This result reinforces the idea that L1 upregulation in the ACC of a subset of ASD subjects might be involved in mechanisms regulating the expression of the host gene.

We then studied whether the genes overlapping upregulated FL L1s (*z* > 3 for L1s) and associated with a negative *z* score (without a specific cutoff on the z for the genes) were associated with neural markers, genes found mutated in ASD and genes highly expressed in the adult brain (see ‘Methods’ section). We call these genes negatively correlated genes. There are about 10 negatively correlated genes in each of the three samples (SRR292614, SRR292620 and SRR292621) accounting for a total of 15 unique genes (Additional file 4: Fig. S3). For each sample, negatively correlated genes resulted enriched for neural markers from single cell studies [65] and for SFARI genes. These enrichments, however, appeared to be a general feature of genes overlapping full-length L1s, the subset of L1s analyzed in this study (*z* score > 25). These genes were not significantly enriched for those highly expressed in the adult brain [64] (Additional file 6: Tables S19–S22). However, five (one-third of the total) negatively correlated genes that are present also in the SFARI database showed the same behavior in all three samples (CADM2, CSMD1, DLG2, DLGAP1, GPHN) (Additional file 4: Fig. S3). The main function of these genes lies in synapse organization and function (Table 2). Examples of mappings for upregulated L1s are provided in Additional file 4: Figs. S4–S6. Figure 4E shows an example of an L1 upregulated in all 3 samples, found within a SFARI gene (DLGAP1) and associated with a negative *z* score. These results suggest that a subset of genes expressed in adult brain might be negatively influenced by the expression of the overlapping intronic FL L1s.

**Table 2:** Description of the 5 genes associated with a negative *z* score and overlapping an upregulated L1 element in all the three samples characterized by pervasive L1 upregulation

Gene name	SFARI score	Gene description (SFARI gene, release 2022 Q3)
CADM2	2	Adhesion molecule that engages in homo- and heterophilic interactions with the other nectin-like family members, leading to cell aggregation. Important for synapse organization, providing regulated trans-synaptic adhesion
CSMD1	2	Weakly expressed in most tissues, except in brain (expressed at intermediate levels in brain, including cerebellum, substantia nigra, hippocampus, and fetal brain). Variants in this gene have been shown to associate with schizophrenia and bipolar disorder
DLG2	2	This gene encodes a member of the membrane-associated guanylate kinase (MAGUK) family. The encoded protein forms a heterodimer with a related family member that may interact at postsynaptic sites to form a multimeric scaffold for the clustering of receptors, ion channels, and associated signaling proteins
DLGAP1	2	The protein encoded by this gene is expressed in the brain, localizes to the postsynaptic density, and interacts with a number of ASD-associated proteins, including DLG1, DLG4, SHANK1, SHANK2 and SHANK3
GPHN	2	This gene encodes a neuronal assembly protein that anchors inhibitory neurotransmitter receptors to the postsynaptic cytoskeleton via high affinity binding to a receptor subunit domain and tubulin dimers. In non-neuronal tissues, the encoded protein is also required for molybdenum cofactor biosynthesis. Mutations in this gene may be associated with the neurological condition hyperplexia and also lead to molybdenum cofactor deficiency

We then calculated the correlation between the transcription level of each expressed FL L1 element with the transcription level of the respective host gene in each dataset (Additional file 5: Tables S15–S18). Interestingly, the percentages of FL L1s whose expression is in negative correlation with the expression of the host gene in Velmeshev data were 50% and 60% for ACC and PFC, respectively, while in the Deneault dataset these percentages were much lower with 3.4% and 0% for iPSC and differentiated neurons, respectively. These results raise the interesting question whether cultured cells are a good system to finely study the expression and the activity of transposable elements.

To validate the observed expression patterns in an independent dataset, we exploited data from a recent study [70], where the authors calculated the coefficient of correlation between the expression of intronic and intergenic L1s and the expression of the overlapping genes in 49 human tissues. The authors stratified L1s into elements expressed in all tissues and in those with a particularly high expression (> 1.5-fold higher) in one tissue compared to all others. Initially, to ease data representation, we grouped the 49 tissues into 11 classes. Our analysis showed that the average coefficient of correlation between intronic tissue-specific L1s and their overlapping genes was substantially lower for brain-derived tissues compared to all other tissues (Fig. 4F). This adds support to the existence of an inverse correlation between intronic L1 and the host genes expression in neurons for a subset of genes. When values for the ungrouped tissues were plotted, results showed differences among areas of the brain. The lower values, suggestive of a stronger negative expression correlation between a subset of L1/gene pairs, were derived from putamen basal ganglia, ACC and cerebellar hemispheres (Fig. 4G). These results support both the idea of regional differences in the brain as well as the existence of a possible relationship specific to the ACC.

Taken together, our results suggest that L1 upregulation might, in some instances, be associated with the downregulation of their host genes. This could happen more frequently in genes important for ASD, with a mechanism yet to be characterized. Of note, the strong enrichments of FL L1-containing genes for neural markers and SFARI classification are interesting and will be important, in future studies, to specifically deepen our knowledge on them.

## Discussion

ASD is a highly heterogeneous group of neurodevelopmental disorders. The identification of common molecular targets is therefore instrumental in defining homogeneous groups of individuals affected by ASD for clinical diagnosis and personalized medicine. Several works determined that transcriptional deregulation affecting both coding and non-coding gene expression occurs in ASD [14–18]. However, the transcription of TEs has often been overlooked with only a few studies showing a general alteration of expression and epigenetic regulation of L1s in ASD [46, 47]. Here, we devoted special attention to the pattern of expression of evolutionarily young FL L1s, since they seem pervasively transcribed and are both a controller and controlled by the epigenetic status of a cell [34, 40, 70, 71]. The main aim of this work was to assess whether L1 expression is altered in ASD brains, in an in vitro model of iPSC and differentiated neurons KOs for several genes known to be directly involved in the etiology of ASD, and in the blood of discordant siblings. Moreover, we aimed at evaluating the impact of L1 expression on the transcription of protein-coding genes.

Our results show that all ASD/KO samples present a moderate positive net number of upregulated L1s. However, a rather consistent increase was evident only in three samples of *postmortem* ASD ACC and in iPSC and differentiated neurons KO for ATRX. These samples showed instances of widespread L1 upregulation, with 30–50% of analyzed L1s presenting significantly higher expression levels compared to controls. ATRX is a SFARI level 1 gene encoding for a protein which contains an ATPase/helicase domain belonging to the SWI/SNF family of chromatin remodeling proteins [52, 68]. ATRX KO cells were previously shown to present increased chromatin accessibility at genomic loci occupied by retrotransposons [68]. By the analysis of public ChIP-seq data for ATRX in human cell lines, we showed a strong enrichment of upregulated L1HS/L1PA sequences in both iPSC and differentiated neurons, suggesting that ATRX loss of function may directly lead to an increased transcription of young FL L1 elements.

Large-scale genome investigations have contributed to the identification of almost one thousand genes putatively involved in ASD [11]. These genes may be arbitrarily divided into two large functional groups: (a) genes which exert a crucial role in synaptic function and (b) genes involved in transcription regulation and/or chromatin remodeling, including ATRX. It is therefore tempting to speculate that mutations in a subset of the latter may directly influence L1 transcriptional regulation, since they are better positioned to have a genome-wide impact on the epigenetic status of chromatin and therefore exert a widespread effect on the transcriptional landscape of cells.

Upregulation seems to occur in a cell-type-specific manner since individuals characterized by the strongest L1 increase in the ACC do not present the same pattern of expression also in the PFC. Furthermore, no changes are observed in the blood of ASD subjects compared with their healthy siblings. However, it is important to note that the analyzed blood dataset is characterized by a rather lower coverage (~ 10–15 million reads of 50 bp per sample) compared to RNA-seq data retrieved from Velmeshev et al. and Deneault et al. (~ 100–150 million reads of 150 bp per sample). Further studies are therefore needed to confront this important issue.

In the study of cell lines, it is interesting to point out that only differentiated neurons KO for ATRX showed a high number of DE genes, although all the KO for ten ASD-related genes showed a certain extent of gene deregulation in iPSC. This was the neuronal sample with the strongest L1 upregulation. This result is consistent with the observation that brain samples characterized by the strongest L1 upregulation are also among the ones with the highest number of DE genes. An increased level of L1 RNA expression in the brain seems thus a biological marker which can be associated only with a subset of ASD cases characterized by the deregulation of a large number of canonical coding and non-coding genes and an increase in intron retention, all features consistent with the idea of a general chromatin dysregulation. The possibility of the existence of two distinct groups of ASD subjects, rather different at the molecular level, is in line with the work by Wong et al. [16] that suggested the existence of two major subgroups of ASDs. While the first subgroup recapitulated the known molecular changes typical of ASD [16], the second one was indistinguishable from control samples in terms of transcriptional and epigenetic alterations.

Most upregulated L1s are intronic, and some of them might be transcribed independently from their host transcripts. In one of the ACC sample characterized by L1 upregulation, a small number of upregulated L1s were hosted in significantly downregulated protein-coding genes. The analysis of an independent dataset [70] added support to the possible existence of an inverse L1/host gene expression relationship for a subset of genes and that this pattern might be a feature of genes with neuronal functions expressed in specific areas of the brain. However, all types of expression patterns were found. An higher number of loci showed a concomitant upregulation of L1s and their host genes, while in other loci increased expression of L1s seemed to have no consequences. The inspection of L1/host gene transcriptional relationship is of crucial importance to understand the effects of L1s on the transcriptional output of the genome in *cis* and warrant further investigations.

We also observed a significant relationship between the number of reads mapped in the introns and the expression of FL L1 (*p* value = 1.4e−08, *r* value = 0.75). We are aware of the fact that an increased number of intronic reads might be responsible for an apparent overexpression of intronic TEs [72]. For polyA + samples, this can be a bias introduced by a differential intron retention. However, when this happens, the apparently upregulated TEs reside in retained introns. In our analysis, we find only a single overlap (out of ~ 800 retained introns) between upregulated TEs and retained introns which suggests that the upregulated L1 might derive from independently transcribed units.

Several models can be proposed on how L1s can regulate the expression of their host genes, influence the differentiation and homeostasis of neurons and be a crucial player in triggering neuronal dysfunction. The function of TEs as regulatory non-coding RNAs is currently under intense investigation. The identification of a large number of DNA/RNA hybrids at L1s loci suggests that TEs might exert their function in *cis* [69]. They can also act by organizing locally chromatin domains within the same *locus* or by recruiting other sequences belonging to different chromosomes. Being capable of interacting with different proteins, they can recruit complexes to specific regions of the genomes. In this context, a recent study showed that L1 RNAs were functionally crucial for binding of Nucleolin-KAP1 complex to its target chromatin, allowing for ESC self-renewal and promoting rRNA synthesis [73]. According to this model, expressed L1 sequences can control and be controlled by the deposition of epigenetic marks and may promote silencing and subsequent re-activation of specific sets of genes during development. Within a gene/L1 pair, L1 expression may give rise to transcriptional interference on the host gene or may guide the establishment of novel epigenetic marks. Given the increasing evidence that epigenetic alteration occurs in ASD [16, 20, 21, 47] and may directly occur at L1s genomic sequences, a direct link between epigenetic control of L1s RNA by regulatory genes mutated in ASD cases and the expression of host genes may be hypothesized.

Our study focuses on L1s fragments longer than 5 kbp, including FL L1s that maintain the potential to retrotranspose. It remains therefore open the possibility that the increased expression of L1s gives rise to uncontrolled retrotransposition and therefore to somatic mosaicism in ASD brains [35, 38, 40]. Recently, ASD brains have been shown to present somatic single nucleotide mutations [74, 75]. A deep analysis of genomic sequences in ASD *postmortem* brains is therefore required to have a full understanding of the molecular consequences of L1s RNA upregulation.

Our results also have relevant clinical implications. If deleterious mutations within ATRX or a defined set of genes with similar roles in L1s transcriptional control are indeed at the basis of the molecular etiology of a subset of ASD subjects, WES-derived data may be used for personalized medicine. Effective medications for the treatment of ASD core symptoms are still lacking. Most of the drug currently in development for ASD is derived from knowledge of genes implicated in monogenic disorders associated with altered neurodevelopmental trajectories and autistic symptoms such as Fragile X, Landau–Kleffner and Rett syndromes [76, 77]. As a consequence, the therapeutic approach to ASD subjects has traditionally focused on associated conditions [76], with poor impact for its core symptoms. These drugs typically target genes associated with synaptic pathways such as dopaminergic and glutamatergic receptors [76]. Interestingly, a recent study revealed that treatment with a low dose of romidepsin restored social deficits in animal models of autism [78]. Romidepsin inhibits the activity of the enzyme histone deacetylase [78], thus restoring the expression of genes involved in neuronal signaling and downregulated in ASD.

Our results have to be considered exploratory and need to be reproduced in bigger cohorts. If validated, they may provide a basis to stratify ASD cases for clinical treatments with drugs modifying the epigenetic status of cells or by interfering with L1 RNA expression. Recent observations on the potential therapeutic use of manipulating TEs in disease conditions in other tissues [79] suggest that molecular tools to interfere with TEs expression could represent a new strategy for the personalized treatment of neurodevelopmental disorders.

## Limitations

Our analyses on *postmortem* brain tissue are mostly limited by the lack of replicates among samples and the small sample size. This is why our analysis should be considered exploratory and needs to be validated in larger cohorts. However, we were able to detect characteristics specific to single samples by restricting the scope of our work to highly expressed L1 elements and by assisting our analyses on tissue-derived samples with in *vitro models* comprising biological replicates. The accurate quantification of TEs expression is a challenging task because of the repetitive nature of their sequence, which complicates the process of mapping sequencing reads to specific *loci*. Furthermore, TE fragments are often embedded in host transcripts and therefore, in most cases, they are unlikely expressed as independent transcriptional units. We aimed at overcoming these challenges by focusing our analyses on only full length, evolutionarily young, L1 elements. We detected a strong upregulation of L1 elements in a subset of ASD cases and in vitro models of ASD which may impact the expression of ASD-relevant genes. It remained to be determined whether this molecular phenotype is strictly linked to pathogenic outcomes or a result of underling alterations at the level of chromatin accessibility.

## Conclusions

The analysis of TEs expression is technically very challenging and caution is needed in the interpretation of results. However, this should not prevent the exploration of the behavior of such elements both at the transcriptional and the genomic level. Much information relative to TEs expression in diseases should be already present in the large amount of data so far collected and, at least in part, made available to the Community. The limited knowledge and lack of widely adopted standard pipelines to analyze TEs often prevent their analysis in the original clinical studies. However, TEs-specific exploration of public dataset has to be considered an important step in exploiting the full potential of genomics.

The importance behind TEs relies on the fact that these elements are revealing a high degree of activity in the brain and their dysregulation appears to be associated with diseases. Given the heterogeneity of neurological diseases and the paucity of studies specifically addressing this issue, it is not surprising that current results, based on small cohorts, seem to be giving contrasting information. Here, we present evidence suggesting that dysregulation of L1s in ASD is not a feature common to all ASD subjects but only to a subgroup of them, clarifying recent observations which proposed dysregulation of L1 as a common feature of ASD subjects. Identifying subgroups of subjects in neurological diseases is crucial and might have therapeutic implications such as being at the basis of stratification of cases for specific clinical treatments informing the choice for specific drugs. The pattern of L1 expression we observed in our analysis could indicate a mechanistic relationship between L1 expression and broader gene expression regulation, or it could represent a marker for widespread expression dysregulation. While our analysis mostly rules out technical biases, results must be taken with the proper care and should be validated in larger cohorts.

## Supplementary Information

**Additional file 1: Table S1.** Sequencing information. **Table S2.** Velmeshev sample information. **Table S3.** Deneault sample information. **Table S4.** Blood sample information. **Table S5.** BAM metrics.

**Additional file 2: Table S6.** Velmeshev TEs consensus expression. **Table S7.** Deneault TEs consensus expression.

**Additional file 3: Table S8.** Velmeshev expressed FL L1. **Table S9.** Deneault expressed FL L1. **Table S10.** DE L1 statistics.

**Additional file 4: Fig. S1.** Results from the Blood cohort. **Fig. S2.** Overlap between different groupings of L1 and expressed genes. **Fig. S3.** Overlaps between negatively correlated genes from the 3 ACC significant samples of the Velmeshev dataset. **Fig. S4.** Reads mapping on L1 intronic to the MARK1 gene in the SRR9292621 sample. **Fig. S5.** Reads mapping on L1 intronic to the MAPK10 gene in the SRR9292620 sample. **Fig. S6.** Reads mapping on L1 intronic to the DLGAP1 gene in the SRR9292614 sample.

**Additional file 5: Table S11.** IRFinder results on single samples. **Table S12.** Expressed L1/gene pairs overlaps. **Table S13.** Upregulated L1/gene pairs. **Table S14.** Upregulated L1/exp gene pairs. **Table S15.** Velmeshev ACC FL L1/host gene correlation. **Table S16.** Velmeshev PFC FL L1/host gene correlation. **Table S17.** Deneault iPSC FL L1/host gene correlation. **Table S18.** Deneault Neurons FL L1/host gene correlation.

**Additional file 6: Table S19**. Anticorrelated genes overlaps. **Table S20.** Anticorrelated genes statistics. **Table S21.** Total FL L1HS-PA overlaps. **Table S22.** Total FL L1HS-PA statistics.

## Data Availability

Most of the analyses have been performed on publicly available data, namely *Velmeshev *et al*.* (PRJNA434002, https://doi.org/10.1126/science.aav8130); *Deneault *et al*.* (PRJNA422099, https://doi.org/10.1016/j.stemcr.2018.10.003); *Valle-Garcìa *et al*.* (PRJNA289924, https://doi.org/10.1080/15592294.2016.1169351); and NIH Roadmap epigenomics mapping consortium (GSM772833, GSM772834, GSM773012, GSM773013, GSM773014, GSM773015; https://doi.org/10.1038/nbt1010-1045).

## Abbreviations

ASD: Autism spectrum disorder
ACC: Anterior cingulate cortex
FDR: False discovery rate
FL: Full length
iPSC: Induced pluripotent stem cell
PFC: Prefrontal cortex
TE: Transposable element
WES: Whole-exome sequencing
