# Supplementary material for: Exploratory analysis of L1 retrotransposons expression in autism﻿
Source: Mol Autism. 2023 Jun 28;14:22. doi: 10.1186/s13229-023-00554-5 (PMC10303858; doi:10.1186/s13229-023-00554-5)
Supplement: Supplementary file 4 — Additional file 4: Fig. S1. Results from the Blood cohort. Fig. S2. Overlap between different groupings of L1 and expressed genes. Fig. S3. Overlaps between negatively correlated genes from the 3 ACC significant samples of the Velmeshev dataset. Fig. S4. Reads mapping on L1 intronic to the MARK1 gene in the SRR9292621 sample. Fig. S5. Reads mapping on L1 intronic to the MAPK10 gene in the SRR9292620 sample. Fig. S6. Reads mapping on L1 intronic to the DLGAP1 gene in the SRR9292614 sample. [file 13229_2023_554_MOESM4_ESM.pdf]

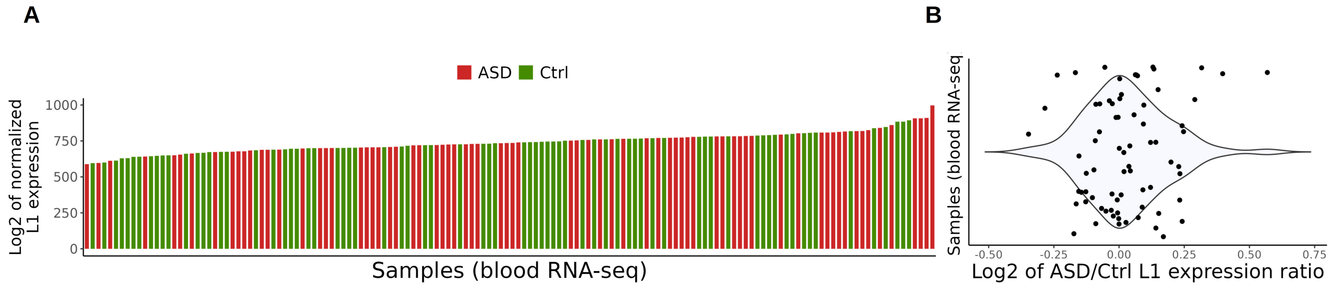

Fig. S1 Results from the Blood cohort. **A** Normalized expression of L1 elements. **B** Espression ratio of L1 elements between ASD and control siblings.

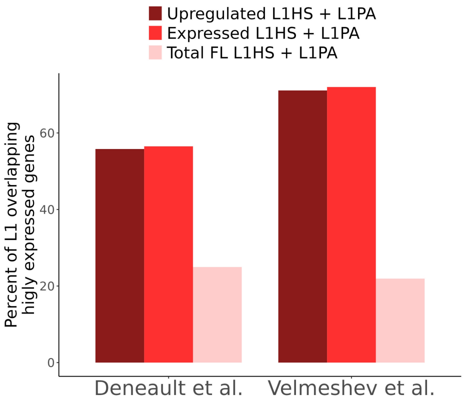

Fig. S2 Overlap between different groupings of L1 and expressed genes.

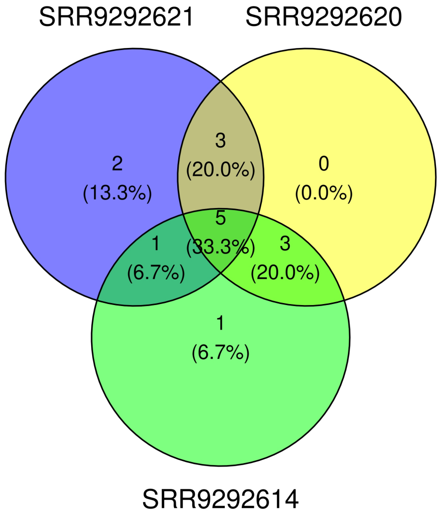

Fig. S3 Overlaps between negatively correlated genes from the 3 ACC significant samples of the Velmeshev dataset.

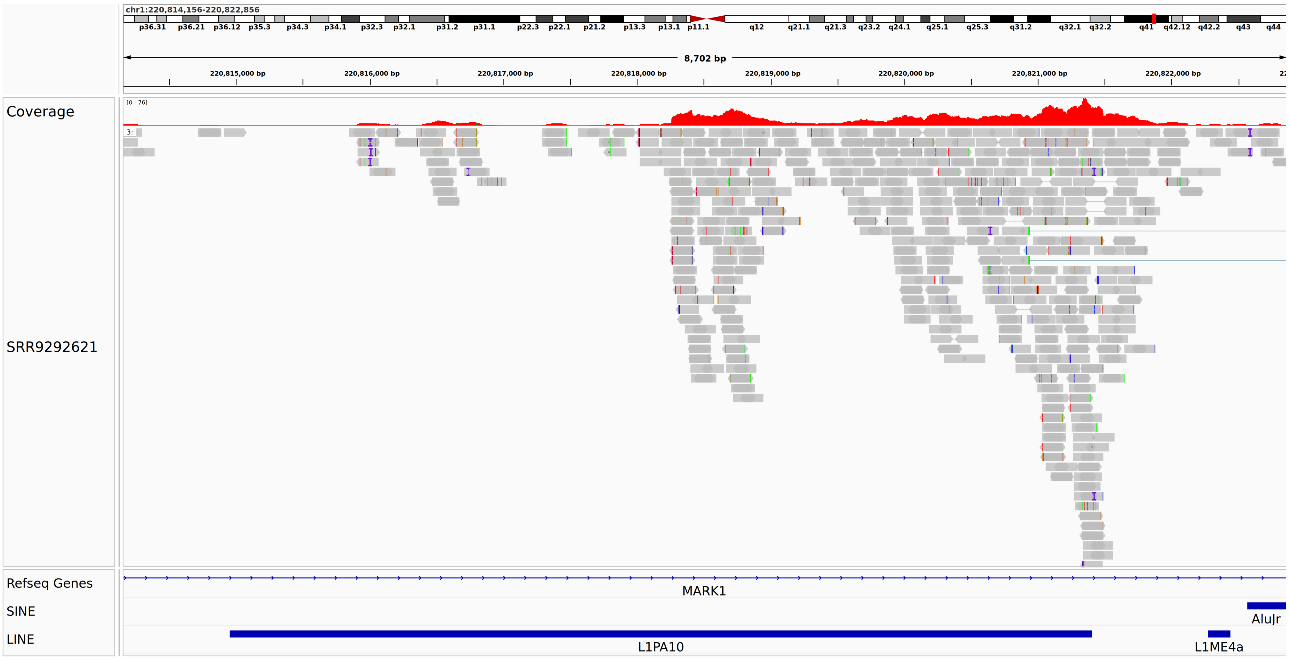

Fig. S4 Reads mapping on L1 intronic to the MARK1 gene in the SRR9292621 sample.

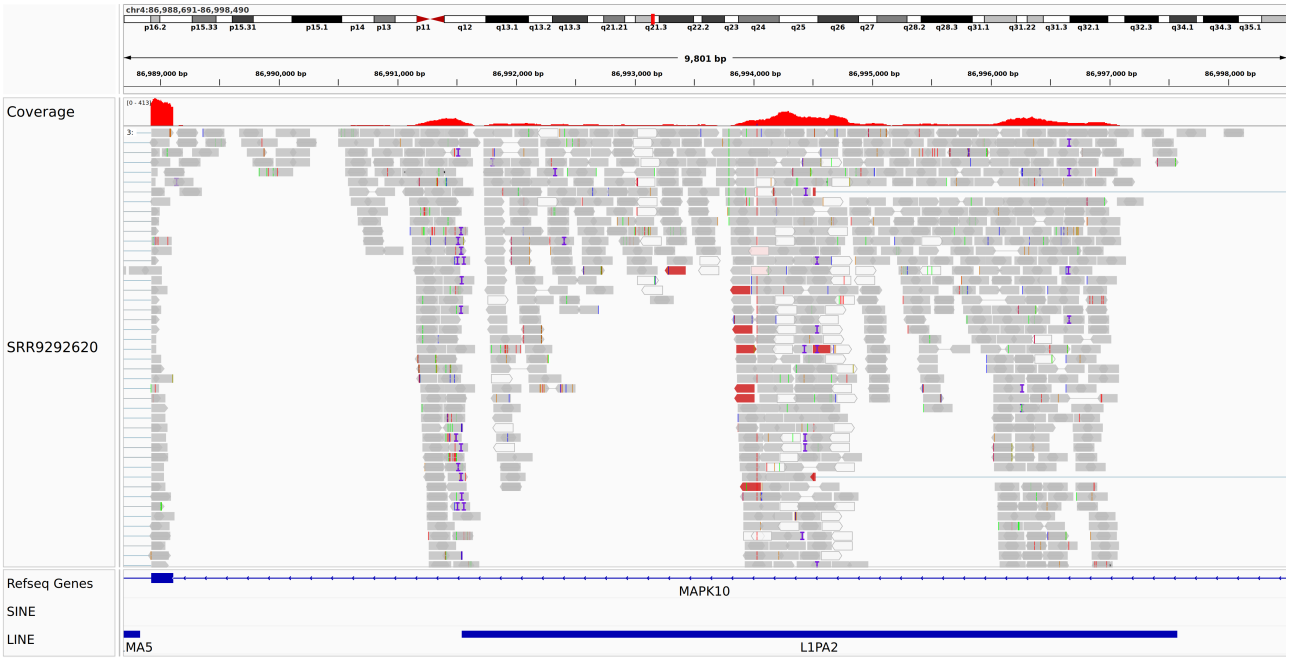

Fig. S5 Reads mapping on L1 intronic to the MAPK10 gene in the SRR9292620 sample.

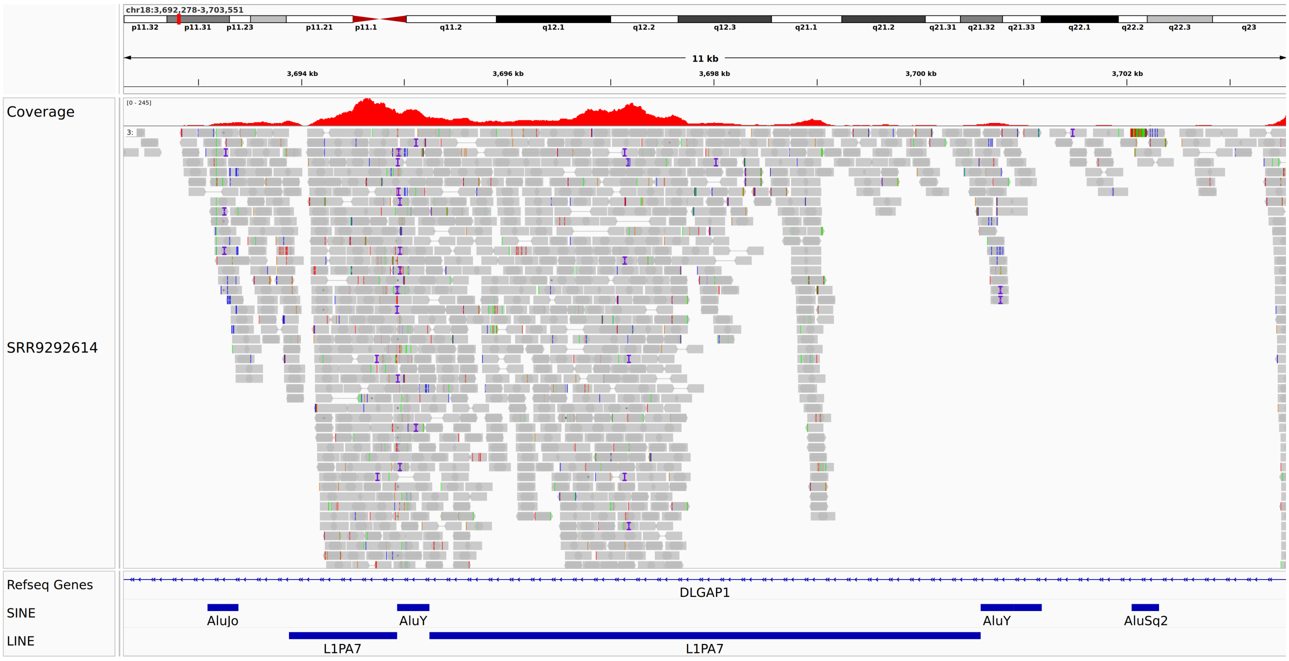

Fig. S6 Reads mapping on L1 intronic to the DLGAP1 gene in the SRR9292614 sample.
